# False memories in cuttlefish

**DOI:** 10.1016/j.isci.2024.110322

**Published:** 2024-07-17

**Authors:** Lisa Poncet, Pauline Billard, Nicola S. Clayton, Cécile Bellanger, Christelle Jozet-Alves

**Affiliations:** 1 Normandie University, Unicaen, CNRS, EthoS, 14000 Caen, France; 2 University Rennes, CNRS, EthoS (Éthologie animale et humaine) - UMR 6552, F-35000 Rennes, France; 3 University of Cambridge, Department of Psychology, Cambridge CB2 3EB, UK

**Keywords:** Behavioral neuroscience, Cognitive neuroscience

## Abstract

Episodic memory is a reconstructive process *per se*: during an event, the features composing it are encoded and stored separately in the brain, then reconstructed when the event’s memory is retrieved. Even with source monitoring processes (e.g., did I see or did I smell it?), some mistakes can occur. These mnemonic mistakes happen especially when different events share several features, producing overlaps difficult to discriminate, leading to the creation of false memories. The common cuttlefish has the ability to remember specific events about what happened where and when, namely episodic-like memory. In order to investigate whether this memory, such as human episodic memory, is based on reconstructive processes, we elaborated a protocol promoting false memory formation. Our results suggest that cuttlefish do form visual false memories, but not olfactory false memories. These memory errors might be the first indication of the presence of reconstructive processes in the memory of cephalopods.

## Highlights

• Episodic-like memory in cuttlefish is based on reconstructive processes
• Visual misleading events impaired memory retrieval ability, creating false memories
• 80% of the cuttlefish remembered a previous event when not misled

## Graphical abstract

**Figure:**
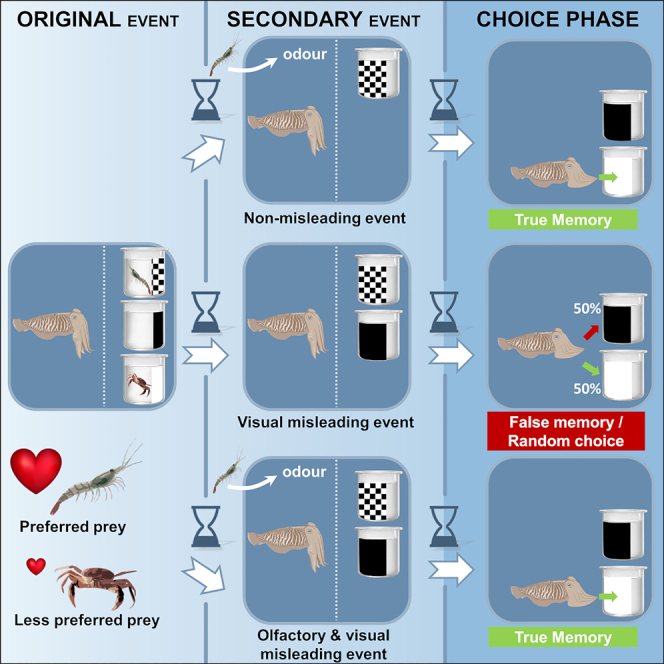

## Introduction

Episodic memory, or the memory of personally experienced events, is a reconstructive process.^1^ Indeed, features of memories are split and stored individually at encoding, and reassembled together at retrieval to recreate the event. Reconstruction is verified by source monitoring processes,^2^ but this monitoring can fail and generate source misattributions from a memory to another memory.^3^^,^^4^ When one or several features of a misleading post-event are misattributed to the memory of an original event, false memories are formed.^5^^,^^6^^,^^7^^,^^8^^,^^9^ They provide a noteworthy indicator of source monitoring failures, providing an indirect way to study source encoding and monitoring processes that lie at the root of the reconstructive memory.

The reconstructive mnemonic processes are rarely explored in non-human animals,^10^^,^^11^^,^^12^ although some studies implicitly investigated reconstruction through features binding,^13^^,^^14^ source memory^14^^,^^15^^,^^16^ or re-ordering memories.^17^ Very few studies on false memories have been conducted in animals^18^^,^^19^^,^^20^^,^^21^; some focusing on false context fear memory,^22^^,^^23^ but none have used false memories to explore the reconstructive nature of episodic memory. In order to explore reconstructive memory abilities, we studied common cuttlefish (*Sepia officinalis*), a cephalopod mollusc. Cuttlefish show episodic-like memory abilities, by remembering what they ate, where and how long ago.^24^^,^^25^ Moreover, it has been recently demonstrated that they possess the ability to retrieve the modality of perception, or source, of an event.^26^ However, in this experiment, they had to retrieve only one contextual feature, while a reconstructive memory would need to retrieve multiple features and assess them in order to discriminate between memories. We thus elaborated an experiment to explore reconstructive processes in cuttlefish by inducing false memories using a misinformation effect paradigm.^27^^,^^28^^,^^29^ As contextual variability, such as the variation of sensory modalities (i.e., seen or smelt) in which information is perceived, has been shown to enhance the likelihood to form false memories in humans,^30^ we used both visual and olfactory misleading information in the experiment. In our experiment, we aimed to test whether cuttlefish would misremember that they had seen shrimps in a tube which was previously empty, after it had been encountered twice concurrently with a tube which previously contained shrimps.

**Figure 1:**
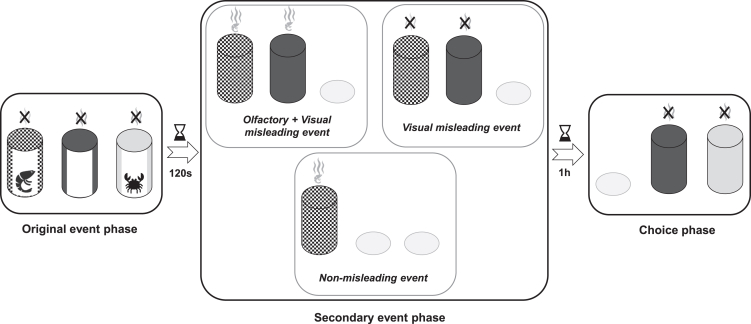
False memory experiment procedure Each condition of the experiment was divided into five phases, with one condition tested per day: the first and last motivational phases (not represented here), and in between, the original event phase, with the content of the three tubes visible, followed by a delay of 120 s; then the secondary event phase, with the content of the tubes not visible: it could be the olfactory and visual misleading event (*OV condition*: a shrimp patterned tube, originally containing shrimp, and an empty patterned tube, with shrimp odor), the visual misleading event (*V condition*: shrimp patterned tube and empty patterned tube with blank water), the non-misleading event (*N condition*: shrimp patterned tube with shrimp odor), all followed by a delay of an hour; then the choice phase, with the cuttlefish allowed to choose between an empty patterned tube and a crab patterned tube. A picture of a glass tube used for the experiment is shown in Figure S1.

To do so, we presented cuttlefish a first event where they visually witnessed different tubes (a netting partition not allowing them to choose one of the tubes): a tube containing a shrimp, their preferred prey, a tube containing a crab, a less preferred prey, and an empty tube (Figures 1 and S1; see STAR Methods for more details). Each tube was associated with a specific pattern. This was followed by a secondary event, where cuttlefish witnessed tubes (a front-back rotation of the tubes allowing them to see the pattern but not the content anymore) without choosing as previously. Cuttlefish were either presented with only a shrimp patterned tube, and shrimp odor (*non-misleading* condition*; N*), or they were misled by presenting an empty patterned tube, in parallel with either visual and olfactory information (a shrimp patterned tube and shrimp odor, *olfactory and visual misleading* condition*; OV*) or only visual information (a shrimp patterned tube with blank water, *visual misleading* condition*; V*). The goal of the misleading conditions was to create an overlap in memory between the content of the shrimp patterned tube and the empty patterned tube making cuttlefish subsequently think that shrimps were present in the empty patterned tube. After a 1-h-delay, cuttlefish could choose between two tubes whose content was not visible: the empty patterned tube, misleadingly associated with shrimps, and the crab patterned tube (non-preferred prey, but true memory; for details see Table S1).

## Results

**Figure 2:**
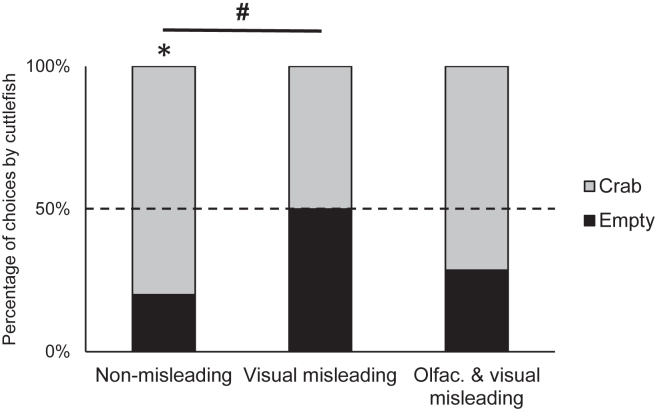
Tube chosen by cuttlefish for each condition in the false memory experiment, each cuttlefish being tested once per condition (*N* = 15 for N condition, and *N* = 14 for V and OV conditions as one cuttlefish did not make any choice; see method details and Figure 1 for a description of the protocol). In the non-misleading condition (*N condition*), cuttlefish significantly favored the crab patterned tube, whereas they did not in to the other conditions. Cuttlefish tended to choose the crab patterned tube more in the non-misleading condition compared to the visual misleading condition (*V condition*), but this difference was not noticeable when compared with the olfactory and visual misleading condition (*OV condition*). Full datasets for the experiment are included in Table S1. The black asterisk indicates a significant difference from chance (i.e., dotted line; binomial test, ∗*p* < 0.05), and the hashtag a trending difference in choices between conditions (binomial GLMER, #*p* = 0.066).

Cuttlefish significantly favored the crab patterned tube over the empty patterned tube in the *non-misleading* condition (binomial test, N: 12 crabs out of 15 choices, *p* = 0.035; Figure 2), whereas no significant preference was observed in the two misleading conditions (OV: 10/14, *p* = 0.180; V: 7/14, *p* = 1). When comparing choices between each condition, a statistical trend was observed between the *non-misleading* and the *visual misleading* conditions (binomial GLMER, SD = 0.827 z-value = 1.837, *p* = 0.066). Latencies to choose did not differ between conditions (Fligner test, *N* = 15, median ± IQR; OV: 55 ± 132.5s; V: 43 ± 74s; N: 53 ± 73.5s; *p* = 0.443), nor when choosing the crab or empty patterned tube (Fligner test, *N* = 15, crab: 53 ± 70s; empty: 36.5 ± 72s; *p* = 0.169), nor with the interaction of conditions and choices (Linear mixed-effect model, *N* = 15, df = 3, F-value = 1.030, *p* = 0.392). It is important to note that two individuals did not choose any tube in one of the two misleading conditions: either the *visual misleading* condition or the *olfactory and visual misleading* condition. During the 10 min of the choice, rather than laying still in their tank, they showed a clear interest for the tubes by looking at them, moving closer and oscillating between them, without making any final choice. These two cuttlefish successfully approached a single tube presented before and after this unsuccessful choice phase, and made a choice in all the other testing conditions.

## Discussion

To sum up, when exposed to misleading information, cuttlefish did not choose the crab significantly more than chance, while they successfully retrieved the location of the crab when they were not exposed to misleading information. While the choices made during the visual misleading condition tended to be statistically different from the choices made during the non-misleading condition, it was not the case when both olfactory and visual information were provided.

Only one tube was presented during the secondary event of the non-misleading condition, while two tubes were used in the two misleading conditions. The presence of two tubes instead of one might have an interference effect during the secondary event. However, it does not explain why cuttlefish formed false memories in the visual misleading condition and not in the olfactory and visual misleading condition.

The false memory experiment likely indicates that cuttlefish were impacted by a misleading event presented after an original event. The similarity of both events might have created an overlap at encoding or retrieval of the memories. There are at least two hypotheses that might explain the absence of a preference for either the crab or the empty patterned tube during the misleading conditions, both being the consequence of the formation of false memories. The first possibility is that cuttlefish relied on chance as they were not able to determine with certainty whether shrimps were or were not previously seen inside the empty patterned tube. This is consistent with the fact that some cuttlefish (while motivated) were not able to make a choice in the misleading conditions. Similarly, in a false memory study with humans,^31^ participants preferred to refrain from answering rather than guessing a response. The other possibility is that some of the cuttlefish were not misled and rightly retrieved the position of the crab, while others may have integrated the misleading information and deliberately chose the empty patterned tube, remembering falsely it previously contained shrimp. Indeed, susceptibility to false memories varies between individuals in humans.^6^ These two explanations are not mutually exclusive: in misinformation effect experiment in humans, it is commonly observed that about a third of the mislead subjects remember a false memory, while another third remember the original event, and the last third rely on guesses.^32^^,^^33^^,^^34^ While an increase in decision time (i.e., choice latencies) could have been expected in the misleading conditions, no difference was found. A potential effect might have been masked by variability within individual responses.

Another point to draw is that when cuttlefish were tested with visual misleading information only, they seem much more impacted than when both olfactory and visual misleading information were provided in comparison with the non-misleading condition (V: 50%, OV: 29% and N: 20% incorrect choices, respectively). Cuttlefish favored the crab patterned tube in the olfactory and visual misleading condition, almost at the same level than in the non-misleading condition. In humans, varying the sensory modalities (auditory or visual) when repeatedly presenting the misleading event usually produce a stronger misinformation effect,^30^ and the same is observed when adding a matching sound to an imagined misleading event.^35^ Three hypotheses can explain our results: 1) the shrimp odor might have lasted until the choice phase and impacted the choices of cuttlefish; or 2) the propensity to create false memories was lower in the olfactory and visual misleading condition than in the visual misleading condition; or 3) exposure to an odor had a resistance effect against the formation of false memory induced by visual misleading information. 1) The first possibility is that the shrimp odor poured in the secondary event phase might have lingered until the choice phase, and this remaining odor could have impacted the choices conducted by cuttlefish. However, this is unlikely given our experimental set-up: there was a delay of 1 h between these two phases which allowed the complete renewal of the water in the tank, thus the remaining odor was expected completely removed before the choice phase. Moreover, even if we consider that they could detect some traces of odor in the choice phase, it should theoretically have increased the propensity to select the empty patterned tube, as increased contextual similarities between the secondary event phase and the choice phase enhance the propensity to form false memories in humans. 2) The second possibility is that the propensity to create false memories was lower in the olfactory and visual misleading condition than in the visual misleading condition. This might be explained by the fact that the overlap between the original and the secondary event was higher in the visual misleading condition as no shrimp odor was available during the original event. As the match was stronger, this might have made cuttlefish more likely to create false memory. This prediction would be in accordance with the encoding specificity principle described by Tulving and Thomson.^36^ However, studies conducted in humans were usually not consistent with this hypothesis, as no difference or opposite results were observed in the literature.^33^^,^^37^ 3) The third possibility is that cuttlefish would not only be resistant to olfactory misleading information, but exposing them to prey odor might have even created resistance against the formation of a visual false memory. It was empirically noticed that during the misleading event phase, after detecting the shrimp odor, some cuttlefish placed themselves in front of the shrimp patterned tube and focused on it, showing no interest in the empty patterned tube nearby. This might have narrowed the scope of cuttlefish’s attention to the shrimp patterned tube during the secondary event, strikingly lowering the salience of the empty patterned tube nearby, making cuttlefish less likely to form false memory.

Nonetheless, some could say that our study does not indicate false memory formation in cuttlefish, but rather simpler memory mechanisms such as familiarity as cuttlefish were exposed twice to the empty tube in the misleading conditions, or even a simple memory loss. We cannot refute that some familiarity mechanisms may be at work in our experiment, however in this case we would have expected the two misleading conditions to affect the choices made in the same order of magnitude in comparison with the non-misleading condition. We also consider memory loss unlikely, as the non-misleading condition indicates that cuttlefish could remember well the position of the crab even when exposed to the shrimp patterned tube between the original event phase and the choice phase.

False memories thus seem to exist in cuttlefish when they are exposed to a visual event sharing features with a previous event. This could be due to the absence of source monitoring processes, but it is unlikely as the memory of the source of an event was previously demonstrated in cuttlefish.^26^ Instead, source monitoring impairment through misinformation effect is more likely.^2^^,^^38^ The observed false memories might be due to binding impairments during encoding or reconstruction errors during retrieval of the memory, whose source was not successfully monitored.^2^^,^^5^

Our study is the first to use false memory to explore reconstructive processes in memory of specific events. Studies on false memories in animals are scarce,^18^^,^^19^^,^^20^^,^^21^ even though they seem promising, as they highlight impairments in source monitoring and reconstructive processes. Moreover, our study is the first to indicate the presence of reconstructive processes in cuttlefish’s episodic-like memory. Reconstruction may be necessary to alleviate cognitive demands, but it may also be used to recombine past scenarios to create and plan for future events,^39^ an ability which still need to be explored in cuttlefish.

### Limitations of study

From a methodological point of view, it was not possible to test the same individuals several times in the same conditions, as multiple replicates per animal would have influenced subsequent choices due to training. Then, our study does not allow us to determine whether or not there are stable interindividual differences in the sensitivity to form false memories. Future studies would benefit from determining these profiles as it will disentangle whether individuals choosing the empty tube did so accidentally (cuttlefish hesitate as they remember where were the crabs, but are not sure whether they had seen or not shrimps in the empty patterned tube) or deliberately (“I have seen shrimps in this tube before”).

## STAR★Methods

### Key resources table

**Table:** 

REAGENT or RESOURCE	SOURCE	IDENTIFIER
**Deposited data**		

Raw data	figshare	https://doi.org/10.6084/m9.figshare.19722328

**Experimental models: Organisms/strains**		

Sepia officinalis	SMEL Synergie Mer Littoral	NA
Sepia officinalis	Centre de Recherches en Environnement Côtier	NA

**Software and algorithms**		

R statistical programming language	R Core Team, 2019	https://www.r-project.org/
lmerTest 3.1 - 3	The Comprehensive R ArchiveNetwork (CRAN)	https://cran.rproject.org/web/packages/lmerTest

### Resource availability

#### Lead contact

Further information and requests should be directed to the lead contact, Christelle Jozet-Alves (christelle.alves@unicaen.fr).

#### Materials availability

This study did not generate new unique reagents.

#### Data and code availability

• All raw data generated in this study have been deposited in Figshare repository and are publicly available as of the date of publication. DOI: https://doi.org/10.6084/m9.figshare.19722328 and see key resources table.
• Any additional information required to reanalyse the data reported in the paper is available from the lead contact upon request.

### Experimental model and study participant details

Cuttlefish rearing and experiments were conducted in compliance with the French regulation for the protection and use of animals in research and the directive 2010/63/EU of the European parliament. Experimental procedures were authorized (#22429 2019101417389263 v2) by the ethical committee of Normandy region (Comité d’Ethique de NOrmandie en Matière d’EXpérimentation Animale, CENOMEXA; agreement number 54).

15 sub-adult cuttlefish (*Sepia officinalis*; 3 to 9 months-old; 3 to 10 cm long, respectively; 8 males, 6 females, 1 undetermined) were trained and tested between September 2021 and March 2022 (False memory experiment). Eggs were collected in the English Channel, cuttlefish hatched and were reared for two months at the SMEL (Synergie Mer et Littoral, Blainville-sur-Mer, France) before being transferred to the rearing facilities of the CREC (Centre de Recherche en Environnement Côtier, Luc-sur-Mer, France) for the following months. Five individuals from the initial group were also included in the complementary experiment ran in March 2022.

### Method details

#### Materials

Cuttlefish were reared individually for the duration of the experiment in a semi-closed system of several cube meters of natural seawater with central filtration, at 17 ± 2°C, under natural light conditions. They were housed in grey squared plastic tanks (20 × 20 × 8 cm) until they reached five centimetres in total length, and then in white rectangular plastic tanks (37 × 28 × 8 cm), enriched with pebbles and plastic algae. They were fed daily with live shrimps (*Crangon crangon*) or crabs (*Hemigrapsus sanguineus*) of suitable size.

Cuttlefish were trained and tested in their home tank. During the experiments, glass tubes (8 cm high x 4 cm in diameter) were used as targets (picture of a glass tube shown in Figure S1). The inside surface of the tube was almost entirely covered with an opaque laminated paper, with a vertical window (about 2 cm wide x 8 cm high) remaining transparent. The paper was printed with various black and white patterns identical on both sides (13 patterns were used; i.e., solid grey, black or white, grey and black checked, black and white striped). By doing so, the cuttlefish could see the content of the tubes by looking through the transparent window, and when the tube was rotated, the content could not be seen anymore. The content of the tubes could not be smelt by tested individuals, as the tubes protruded above water, preventing the water inside the tubes to mix with the tank water. When several tubes were used in parallel, each tube presented a different pattern. To prevent immediate access to the tubes, a green rigid plastic netting was placed between the tubes and the cuttlefish, about ten centimetres away from the tubes. The shrimp odor, poured in the tank during the experiments, was made of 250 mL taken from of a bucket which contained ten live shrimps (*Crangon crangon*) per liter of seawater during at least 10 min. All the tubes were thoroughly cleaned between each phase.

#### Training

Firstly, cuttlefish were trained to spontaneously approach a glass tube covered with paper with a pattern randomly chosen for each trial (among 13 different patterns). Three to five training trials were conducted each day. To do so, a tube was placed in their tank, and after a delay of 15–120 s (shorter at the beginning of the training, longer at the end), a shrimp on a fishing line was put next to the tube, so the cuttlefish could catch it. After the cuttlefish spontaneously approached the tube in less than 120s three times within the same day, the next step of the training was conducted.

Secondly, the cuttlefish learnt to choose between two tubes, one containing a shrimp and one containing a crab. To do so, a first tube was put and the cuttlefish could see the prey inside it (a shrimp or a crab) for 15 s before the tube was turned, hiding the prey. A second tube was presented in the same way, containing the other prey type. A netting, placed between the cuttlefish and the tubes, prevented the access to the tubes. It was removed after at least 5 s and the cuttlefish could choose between the two tubes. The cuttlefish was rewarded with the prey corresponding to the chosen tube, and it was considered a successful trial. If the cuttlefish did not choose after 3 min, a shrimp was put next to the tube containing the shrimp, and it was considered a failed trial. After 9 successful trials in less than 15 consecutive trials, a third tube was added, which did not contain any prey, for at least three consecutive successful trials.

Thirdly, a preference test was conducted: a crab and a shrimp at the end of a fishing line were presented in front of the cuttlefish at the same time, and the seized prey was noted. After twelve successive presentations, preference for one prey was assessed using binomial tests. The false memory experiment started the day after the completion of the preference test.

All tested cuttlefish succeeded the training phase 1 (i.e., approaching a tube to get food). They learnt to spontaneously come next to the tube in 119 ± 57 trials. They all learnt to choose a tube out of two in 10.7 ± 2.2 trials (mean ± SD; training phase 2). None choose the empty tube instead of the shrimp or the crab tube during the three trials of the training phase 3. All tested cuttlefish preferred shrimps over crabs: 10 to 11 choices of shrimps out of 12 successive trials (mean ± SD: 10.3 ± 0.5; binomial test, *p* < 0.039; see Table S1).

#### False memory experiment procedure

The false memory experiment was constituted of three different testing conditions: two misleading and one non-misleading conditions (Figure 1). Each cuttlefish was tested once in each of the three conditions. The misleading conditions were designed to promote the formation of false memories of shrimp presence (preferred prey), by using only the visual modality (V) or using both olfactory and visual modalities (OV). The non-misleading condition (N) was designed to control cuttlefish ability to remember the position of the crab (non-preferred prey). Cuttlefish were tested on each condition, one condition per day, in a randomized order.

All conditions followed the same procedure. Five phases could be distinguished: a first motivational phase, an original event phase, a secondary (misleading or non-misleading) event phase, a choice phase, and a last motivational phase.

The first motivational phase was conducted each day to assess and maintain the motivation of cuttlefish to reach for a tube to obtain a reward. During this phase, an opaque tube (with a pattern different from the ones used in the following phases) was placed in the tank and the cuttlefish obtained a shrimp by choosing the tube in less than 120 s. Throughout the experiment, we considered that cuttlefish made a choice when they oriented their body and eyes toward one tube while staying at a distance lower than ten centimetres from the tube, or by circling around it, for at least 10 s. If the motivational procedure failed a first time, it was done a second time a few minutes later, and if it failed a second time, the rest of the experiment was rescheduled until the next day (experiment was rescheduled one time for three cuttlefish).

In the original event phase, three tubes were used. They were covered with three different patterns (out of the 13 used during the training and testing) which changed randomly between conditions. One tube contained a shrimp (*Crangon crangon*), the preferred prey of cuttlefish, one tube contained a crab (*Hemigrapsus sanguineus*), a less preferred prey, and one tube was empty. The phase started by placing a netting ten centimetres away from one side of the tank (i.e., to make cuttlefish able to see the tubes but not to choose one of them). The three patterned tubes were placed behind the netting, next to each other. They were rotated one after the other, in a random order, so that their content was visible for the cuttlefish for 15 s before being hidden again. Tubes were removed all at once and a delay of 120 s began.

Next, the secondary event phase was conducted: one (non-misleading condition) or two (misleading conditions) tubes were presented, with their content not visible but their pattern clearly visible. 250 mL of blank sea water or sea water with shrimp odor was gradually poured behind the single tube or between the two adjacent tubes in front of the water inflow. For the non-misleading condition, a shrimp patterned tube (i.e., a tube with a shrimp during the original event) was presented with shrimp odor. For the olfactory and visual misleading condition, the shrimp and the empty patterned tubes (tubes with a shrimp or empty during the original event, respectively) were presented with shrimp odor. For the visual misleading condition, the shrimp and the empty patterned tubes were presented with blank sea water. The tubes and netting were removed after 60 s and a delay of 1 h began.

Next, during the choice phase, the crab patterned tube and the empty patterned tube were simultaneously placed in the tank (without the netting partition). Cuttlefish could choose one of the tubes, and they were not rewarded whatever the choice made. If no choice was made within 10 min, tubes were removed from the tank.

At last, the last motivational phase was conducted similarly to the first motivational session, to assess and maintain the motivation of cuttlefish to choose for a tube to obtain a reward. In this motivational phase, if the cuttlefish did not choose the opaque tube (which presented the same pattern as in the first motivational phase) within 120 s, it was still rewarded by placing a shrimp next to the tube.

### Quantification and statistical analysis

All choices in the false memory experiment were recorded using a video camera (Sony Handycam FDR-AX53 4K). Choices as well as latency before choice were scored by one experimenter (L.P.). Latency (an indicator of decision time) was measured from the time tubes were put in the tank to the time the cuttlefish approached less than ten centimetres away from the chosen tube. The distance was estimated using reference points on top of the home tank. The experimenter scored the choices of each cuttlefish twice to verify intra-observer reliability: the first scoring was unblinded and made directly during the experiment, while the second scoring was conducted several months after the end of the experiment, and the experimenter was blinded to both condition and tube pattern. The intra-observer reliability subsequently obtained was of 100%.

Choices and latencies were analyzed using R software (version 3.6.3, R Core Team, 2019^40^). Choices made during the false memory experiment were analyzed using binomial tests and a Generalized Linear Mixed Model (GLMM, package lmerTest) with a binomial distribution. We included the choice cuttlefish made as the response variable, the condition as the fixed effect and the cuttlefish as the random intercept effect. Latencies were analyzed using non-parametric Fligner tests and Linear Mixed Models (package lmerTest), with the duration of the choice as the response variable, the interaction between choice and condition as the fixed effect, and the cuttlefish as the random intercept effect.
