# Supplementary material for: False memories in cuttlefish
Source: iScience. 2024 Jul 17;27(8):110322. doi: 10.1016/j.isci.2024.110322 (PMC11384069; doi:10.1016/j.isci.2024.110322)
Supplement: Document S1. Figure S1 and Table S1 [file mmc1.pdf]

iScience, Volume *27*

Supplemental information

False memories in cuttlefish

Lisa Poncet, Pauline Billard, Nicola S. Clayton, Cécile Bellanger, and Christelle Jozet-Alves

Table S1: Choices made by each cuttlefish in the false memories experiment, summarized by Condition in Figure 2. Conditions are abbreviated as N: non-misleading, V: visual misleading, OV: olfactory and visual misleading. Numbers within parentheses in the food preference column correspond to the number of times a prey item was chosen during the food preference test. Position of the tubes is presented from left to right, with C: Crab tube E: Empty tube, S: Shrimp tube, with the chosen tube in bold.

Individual	Sex	Age at test (months)	Food preference	Condition N		Condition V		Condition OV	
				Choice	Tubes position	Choice	Tubes position	Choice	Tubes position
**1**	M	5	Shrimp (11/12)	Crab	E **C** S	Empty	C S **E**	Empty	S **E** C
**2**	F	5	Shrimp (10/12)	Crab	**C** E S	Crab	E S **C**	Crab	E **C** S
**3**	F	5	Shrimp (11/12)	Crab	**C** E S	Empty	**E** C S	Empty	**E** S C
**4**	M	5	Shrimp (10/12)	Empty	C **E** S	Empty	**E** C S	Crab	S E **C**
**5**	NA	5	Shrimp (10/12)	Crab	**C** E S	Empty	C S **E**	Empty	**E** S C
**6**	F	5	Shrimp (10/12)	Crab	**C** E S	Empty	C S **E**	Crab	E **C** S
**7**	M	5	Shrimp (10/12)	Crab	**C** S E	Crab	S **C** E	Crab	**C** S E
**8**	M	5.5	Shrimp (10/12)	Crab	S **C** E	Crab	E **C** S	None	C S E
**9**	F	5.5	Shrimp (10/12)	Empty	**E** S C	Empty	C S **E**	Crab	**C** S E
**10**	M	5.5	Shrimp (11/12)	Crab	E S **C**	Crab	**C** S E	Crab	S **C** E
**11**	F	6.5	Shrimp (10/12)	Empty	S **E** C	Crab	E S **C**	Crab	S **C** E
**12**	M	7	Shrimp (10/12)	Crab	S E **C**	Crab	S **C** E	Crab	**C** E S
**13**	M	7	Shrimp (11/12)	Crab	S **C** E	Empty	C **E** S	Crab	S E **C**
**14**	M	7.5	Shrimp (10/12)	Crab	S E **C**	None	C E S	Crab	S E **C**
**15**	F	8	Shrimp (10/12)	Crab	E **C** S	Crab	S E **C**	Empty	C **E** S

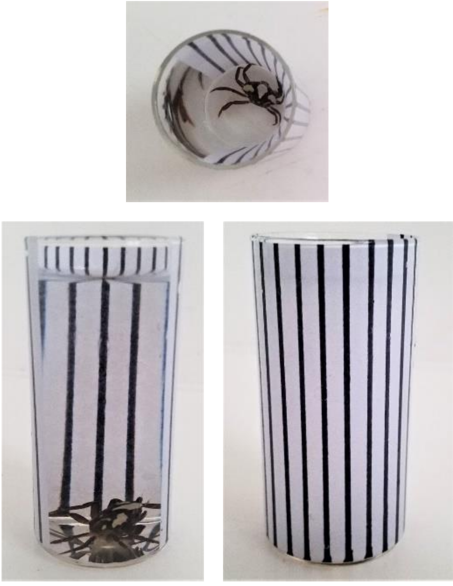

Figure S1: Glass tube used during the false memory experiment described in Figure 1. The tube was partially covered with patterned paper, so the prey inside could be visible through a window then became invisible when the tube was rotated.
